# Foodborne Botulism Caused by *Clostridium botulinum* Subtype A5(b3) by Self-Packaged Vacuum Spicy Rabbit Heads

**DOI:** 10.3390/microorganisms13071662

**Published:** 2025-07-15

**Authors:** Wen Cui, Chuanmin Ma, Ming Liu, Yan Li, Lin Zhou, Yuwen Shi, Xuefang Xu, Hui Liu

**Affiliations:** 1 Jinan Center for Disease Control and Prevention Affiliated to Shandong University, Jinan 250021, China; 2 Huaiyin District Center for Disease Control and Prevention, Huaiyin District, Jinan 250021, China; 3 National Key Laboratory of Intelligent Tracking and Forecasting for Infectious Diseases, National Institute for Communicable Diseases Control and Prevention, Chinese Center for Disease Control and Prevention, Changping, Beijing 102206, China

**Keywords:** foodborne botulism, *Clostridium botulinum*, BoNT, spicy rabbit head, subtype A5(b3)

## Abstract

Botulism is a severe muscle paralysis disease mediated by the botulinum toxin. Here, we reported a foodborne botulism case caused by *Clostridium botulinum* subtype A5(b3) from self-packaged vacuum spicy rabbit heads. Treatment for this case was delayed due to misdiagnosis and insufficient diagnostic capacity in three hospitals, which resulted in progressive clinical deterioration, and eventually, the patient was transferred to Shandong Public Health Clinical Center for specialized therapy. The case was suspected as foodborne botulism by the Qilu Medical-Prevention Innovation Integration pathway and multi-disciplinary consultation. An epidemiological investigation and laboratory confirmation revealed that the botulinum neurotoxin originated from vacuum-packaged spicy rabbit heads distributed via interprovincial cold chain logistics. After treatment with botulism antiserum, the patient’s condition significantly improved, and they were discharged after recovery. We revealed that this foodborne botulism outbreak was caused by the *Clostridium botulinum* A5(b3) subtype from food by whole-genome sequencing and SNP typing. All the strains belonged to Group I carrying the botulinum neurotoxin gene classified as the ha cluster. Toxin A was confirmed by MBA and other methods, while toxin B was non-functional due to the truncated bont/B gene. Other virulence genes and antibiotic resistance genes were also detected. Our findings indicate that self-packaged vacuum meat products represent an emerging risk factor for botulism transmission when stored improperly. Importantly, the recurrent misdiagnosis in this case underscored the urgent need to enhance the training of healthcare professionals in medical institutions to improve the diagnostic accuracy and clinical management of botulism.

## 1. Introduction

*Clostridium botulinum* (*C. botulinum*) is an anaerobic bacterium mostly producing a potent botulinum toxin (BoNT), which causes severe disease with a high mortality rate [1,2,3,4]. The botulinum toxin comprises a heavy chain (100 kDa) and a light chain (50 kDa). As a zinc metalloprotease, its light chain can access the cytoplasm of nerve cells, where it specifically cleaves the soluble N-ethylmaleimide-sensitive factor attachment protein receptors (SNARE) associated with the release of the neurotransmitter acetylcholine, thereby effectively inhibiting its release [5,6,7]. Meanwhile, the botulinum toxin can bind to the presynaptic membrane, obstructing the opening of calcium channels in the nerve cell membrane [8,9]. This ultimately leads to fatal flaccid muscle paralysis. BoNTs can be classified into seven types (A–G types). Types A, B, E, and F can cause poisoning in humans, while types C and D mainly cause disease in animals [10,11,12].

Five types of botulism are commonly identified in human cases, including foodborne botulism, infant botulism, wound botulism, iatrogenic botulism, and inhalational botulism [4,13]. Foodborne botulism is a poisoning caused by the consumption of food containing the botulinum toxin, and as little as 30 ng of the neurotoxin is enough to cause illness or even death [14]. In Canada and France, the most common food products for foodborne *C. botulinum* poisoning are home-made traditional processed fish products and canned fish [15,16]. Fermented homemade items such as stinky tofu and noodle sauce have primarily caused foodborne botulism in China. The highest incidence of botulism in China is in the northwestern region, with Xinjiang province having the highest incidence rate [17,18]. Studies have demonstrated that the predominant BoNT serotypes were type A in China. Foodborne botulism cases mainly occur from June to August due to high temperature and high humidity, which is suitable for the reproduction of *C. botulinum* and the production of toxins [17].

In this study, we reported a case of foodborne botulism that worsened due to misdiagnosis in multiple hospitals. To clarify the cause of poisoning and provide relevant support for clinical treatment, a rapid epidemiological investigation was conducted on a confirmed case and their family members by the Huaiyin District CDC and Jinan CDC. Suspicious food and fecal samples from the case were collected for nucleic acid testing. Meanwhile, whole-genome sequencing was carried out on the strains. Combined with clinical symptoms, epidemiological investigation results, and laboratory test results, it was confirmed as a foodborne botulism event caused by BoNT/A5 (b3). The patient’s condition improved significantly after treatment with botulinum toxin antiserum, and he was discharged from the hospital.

## 2. Epidemiological Investigation

On 16 December 2023, a 53-year-old man presented to a local hospital with symptoms of fatigue, dizziness, diplopia, and swelling in his right eye. Magnetic resonance imaging (MRI) indicated the presence of a cerebral infarction. After four days, the patient was transferred to another hospital with a symptom of stiff base of the tongue. The provisional diagnosis upon admission was “Cerebral infarction?”. Subsequently, the patient underwent a series of tests and treatments related to cerebral infarction management, including neuroimaging studies and antithrombotic therapy. Unfortunately, the patient’s neurological status progressively deteriorated.

**Figure 1:**
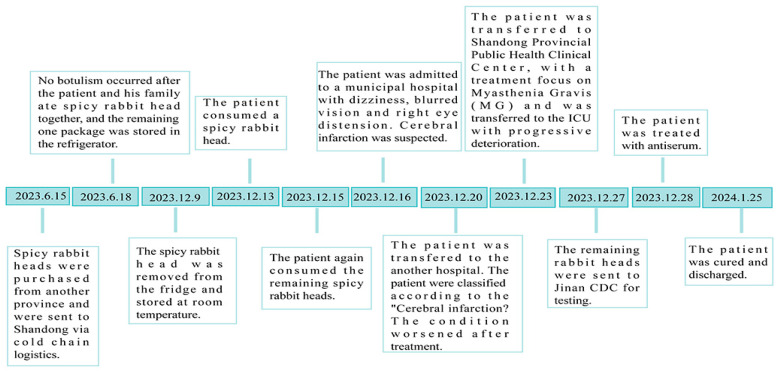
Timeline of symptom onset and epidemiological investigation in patient.

On 23 December, the patient was transferred to Shandong Provincial Public Health Clinical Center for hospitalization, with a treatment focus on myasthenia gravis (MG). A day later, the patient exhibited symptoms of dysphagia and subsequently received prophylactic immunoglobulin therapy. Serum AchR-Ab test results were negative, precluding a definitive diagnosis of MG. Electromyographic findings failed to confirm the diagnosis of Guillain–Barré syndrome (GBS). The lack of conclusive diagnostic evidence led to a deterioration in the patient’s condition, necessitating his transfer to the intensive care unit (ICU). On 27 December, the Shandong Provincial Public Health Clinical Center launched the Qilu Medical-Prevention Integration Innovation Mechanism, which convened a multidisciplinary consultation involving with Qilu Hospital of Shandong University, the Jinan Center for Disease Control and Prevention, and Shandong Provincial Hospital. Based on expert assessments, the case was preliminarily suspected as foodborne botulism. The Jinan Center for Disease Control and Prevention promptly carried out an epidemiological investigation to pinpoint the causative agent, thereby informing clinical management strategies. This investigation revealed that the patient had consistently shared meals with family members before symptom onset. The only dietary deviation was the consumption of a spicy rabbit head on 15 December at noon. Through dietary history analysis, the spicy rabbit head was identified as the potential risk food. We collected patients’ feces and spicy rabbit heads for the detection of BoNT and *Clostridium botulinum*. Subsequent trace-back investigations revealed that this vacuum-packed spicy rabbit head had been purchased online and delivered to Shandong via cold-chain logistics (Figure 1).

The preliminary analysis indicated a suspected foodborne disease outbreak caused by botulinum toxin contamination in the spicy rabbit head. The Jinan CDC conducted testing on collected samples, including suspected food specimens, patient’s feces, and serum. Additionally, whole-genome sequencing (WGS) was performed on the strains to enable a more precise identification and comprehensive analysis.

## 3. Materials and Methods

### 3.1. Isolation of Clostridium botulinum

The process of enrichment culture and strain isolation were carried out according to national standards for food safety (microbiological test of food and *Clostridium botulinum* and botulinum toxin test GB 4789.12-2016 [19]). Fecal and spicy rabbit head sample dilutions were aspirated and inoculated into four cooked meat culture tubes and two TPGY tubes, which were then placed in 36 °C and 28 °C, respectively, for anaerobic incubation over a five-day period. The turbidity, gas production, and meat residue digestion of the culture tube were meticulously observed. The suspected enrichment solution was inoculated onto egg yolk agar, followed by anaerobic incubation at 36 °C for 48 h. Any suspicious colonies were selected for gram staining for microscopic examination, real-time quantitative PCR, a mouse bioassay, and whole-genome sequencing (WGS).

### 3.2. DNA Extraction and Real-Time Quantitative PCR Typing

The fecal samples of the patients and spicy rabbit heads were diluted with sterile PBS at a ratio of 1:100 for DNA extraction. *C. botulinum* colonies obtained from feces and spicy rabbit heads were resuspended in 200 μL PBS for DNA extraction. A volume of 200 μL of the sample was added to the sample tube from the BD MAX Exk TNA-2 kit (BD Biosciences, San Jose, CA, USA). The instructions from the BD MAX Exk TNA-2 nucleic acid extraction kit were followed to perform rapid nucleic acid extraction for 30 min. Subsequent quantification was achieved by a Qubit fluorescence spectrometer (Thermo Fisher Scientific, Waltham, MA, USA).

The extracted DNA from fecal samples, spicy rabbit heads, and *C. botulinum* colonies was tested for the presence of botulinum toxin genes A, B, E, and F using a *C. botulinum* toxin-producing gene test kit (Shengkeyuan, Beijing, China). The reaction volume was set to 25 μL. Thermal cycling conditions consisted of a heat treatment at 50 °C for 2 min and a pre-denaturation step at 95 °C for 3 min, followed by 40 cycles at 95 °C for 3 s and 55 °C for 30 s. Real-time quantitative PCR was conducted on the Thermofisher QuantStudio5 instrument (Thermo Fisher Scientific, Waltham, MA, USA).

### 3.3. Toxin Detection of Patient Samples

The serum obtained by centrifuging the blood samples of the case at 4000 rpm for 5 min was used for toxin detection. The patient’s fecal matter and spicy rabbit heads were diluted with sterile PBS at a ratio of 1:100 for toxin detection. Botulinum toxin types were co-assayed by lanthanide high-sensitivity fluorescence immunochromatography (HSFICA) (China, Bo Rui Biology) and colloidal gold methods (Beijing Jinhao Pharmaceutical, Beijing, China) for serum samples, patient fecal matter, and spicy rabbit heads. The HSFICA method detects the botulinum toxin using an automatic fluorescence immuno-chromatographic analyzer, calculating the sample concentration based on the standard curve and determining the results (positive or negative) [20]. The colloidal gold method for botulinum toxin detection involves adding 200 μL of the sample and visually determining the results within 15 min [21].

### 3.4. Assessment of BoNT Toxin by Mouse Bioassay Experiments

Samples, including spicy rabbit heads, patient feces, suspected enrichment cultures, and a TPGY culture of isolated *C. botulinum*, were carried out by the Chinese Center for Disease Control and Prevention (CDC). The presence of botulinum toxin in these samples was confirmed via mouse bioassay experiments, following the national standard GB/T 4789.12-2016. All animal experiments were performed in accordance with the Chinese Regulations on the Administration of Laboratory Animals and were approved by Laboratory Animal Ethics Committee of the Chinese Center for Disease Control and Prevention (name of research project: Detection of botulinum toxin; approval code: 2023-041; approval date: 18 October 2023).

### 3.5. Analysis of Whole-Genome Sequencing (WGS) and Genomic Characteristic Collinearity of C. botulinum Strains

We identified three suspected *Clostridium botulinum* strains from patient (JNPF1) and spicy rabbit head samples (JNSRH1, JNSRH2). To obtain comprehensive genomic data, we performed whole-genome sequencing (WGS) on the Illumina HiSeq 2500 platform (San Diego, CA, USA) using paired-end 150 bp (PE150) reads. After quality control with fastp [22], high-quality reads were assembled de novo using multiple assemblers and refined with CISA and GapCloser to improve contiguity and completeness. For core single-nucleotide polymorphism (SNP) analysis, the assembled contigs from all 21 isolates, including the 18 publicly available genomes from NCBI, were aligned to the *C. botulinum* reference genome GCF_000063585.1 using Snippy (v4.6.0). SNP alignments were merged using snippy-core to generate a core genome alignment, excluding non-core regions, indels, and low-quality sites. A maximum likelihood (ML) phylogenetic tree was constructed based on this alignment using FastTree (v2.1) under the generalized time-reversible (GTR) model. Additionally, to investigate the neurotoxin of the strain, the *bont* gene sequences of the strains were extracted and compared with the BoNT toxin sequences of the A1-A8 subtypes to construct a phylogenetic tree using the neighbor-joining method (MEGA11.0.13).

We extracted *bont* gene clusters from the JNPF1 genome and downloaded three different *bont* gene clusters for A5 from the GenBank database (accession numbers: IBCA94-0216; H04402065; A661222). A collinearity analysis of toxin gene cluster arrangements was performed by Easyfig software (version 2.2.5, Brisbane, Australia).

## 4. Results

### 4.1. Detection of BoNT and BoNT Genes

Testing toxin genes in the patient’s feces and the spicy rabbit head by the Jinan CDC indicated the presence of botulinum toxin genes of types A and B. Subtype A BoNT was detected in serum by HSFICA and colloidal gold methods. BoNT A was also found in patient fecal matter and spicy rabbit heads by the mouse bioassay (MBA), HSFICA, and colloidal gold tests. The BoNT A production of strains was confirmed by MBA as well (Table 1). Based on the symptoms presented and the toxin gene results, the hospital administered a botulinum antitoxin to the patient.

### 4.2. Isolation and Identification of C. botulinum

Following Gram’s staining, we identified the strains of suspected botulinum bacteria colonies from the patient (JNPF1) and spicy rabbit heads (JNSRH1, JNSRH2) (BioSample: SAMN48975262; accession numbers: JBPCFB000000000). These were observed to be Gram-positive bacillus. The toxin genotypes of the strains were shown to be genotypes A and B using Q-PCR (Table 1). This is consistent with the genotypes found in the samples.

**Figure 2:**
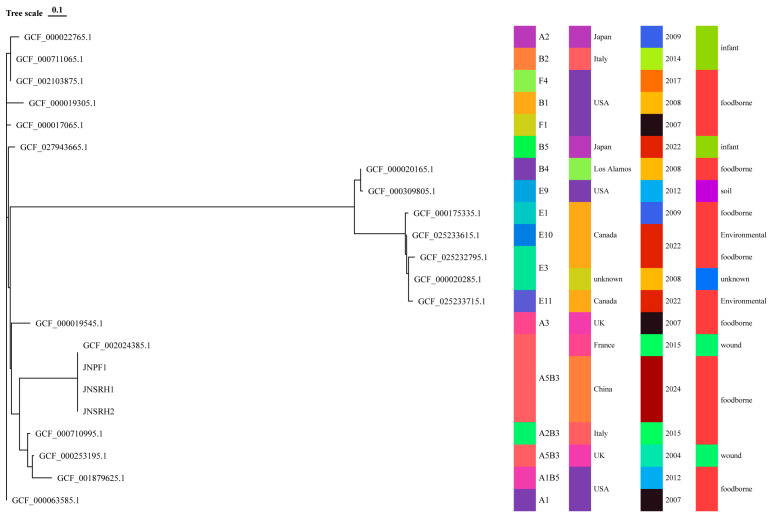
Genomic characteristics of *Clostridium botulinum* strains: phylogenetic tree of *Clostridium botulinum* strains based on coreSNP analysis.

The genome sizes of JNPF1, JNSRH1, and JNSRH2 were 3,751,083 bp, 3,955,489 bp, and 3,763,853 bp, respectively. The GC contents were 27.19%, 27.97%, and 27.92%, respectively. Upon analysis, the core SNP differences among the strains were determined to be less than five, suggesting that these samples were from the same bacterial strain. Phylogenetic analysis further confirmed this hypothesis by demonstrating that the strains of *C. botulinum* in this study were situated on the same evolutionary clade, indicating a shared origin. We found that the *C. botulinum* strain belonged to Group I and were in the same clade as GCF_002024385.1, GCF_000710995.1, GCF_000253195.1, GCF_001879625.1, and GCF_000019545.1 strains from Group I (Figure 2).

### 4.3. Botulinum Neurotoxin Types and MLST Typing

**Figure 3:**
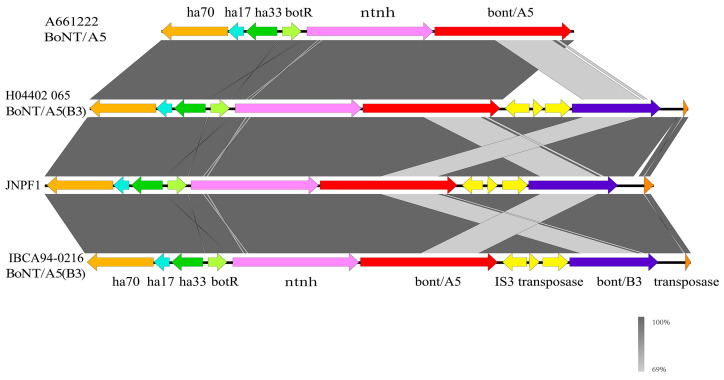
Comparison of the *bont* gene collinearity analysis of in JNPF1 and three A5 genotype *Clostridium botulinum* genomes (the gradient of gray levels represents different BLAST identity values).

**Figure 4:**
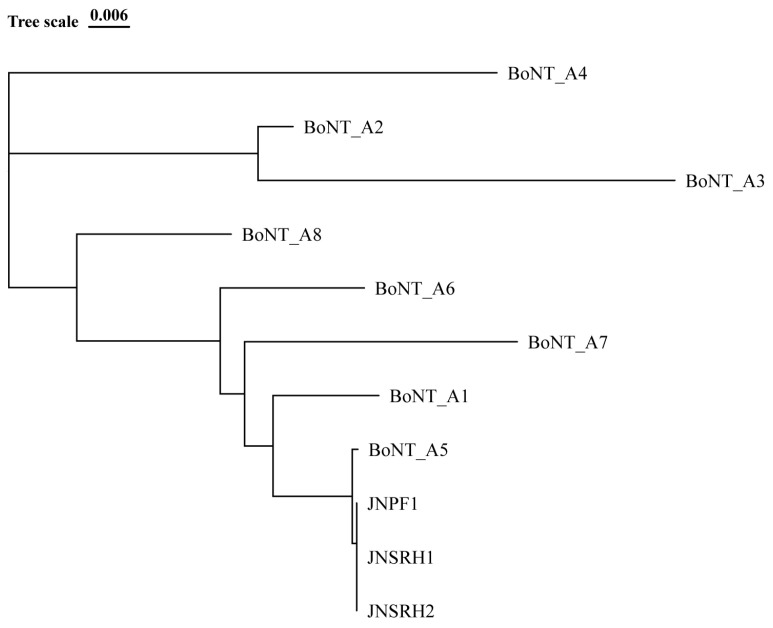
Phylogenetic tree of the *bont* gene nucleic acid sequences.

Using Easyfig (version 2.2.5, Australia) software for analysis revealed that *bont*/A5 and *bont*/B3 in the JNPF1 gene cluster was located in a hemagglutinin complex (ha), with three is3-family transposases existing upstream of the truncated *bont*/B gene. The truncated *bont*/B gene is the nucleotide sequence lacking the light chain encoding the toxin. Upon conducting a BLAST (https://blast.ncbi.nlm.nih.gov/Blast.cgi, accessed on 10 January 2024) comparison, this sequence exhibited the highest similarity to the *bont*/B3 isoform. The *bont* gene cluster of JNPF1 has the same arrangement and position as both BoNT/A5(B3). In conclusion, the correlation analysis confirmed that the JNPF1 strain belongs to the BoNT/A5(B3) subtype (Figure 3). *C. botulinum* JNPF1 is classified under ST16 by the PubMLST online website analysis (https://pubmlst.org/organisms, accessed on 10 January 2024), consistent with GCF_002024385.1 (A5(b3)). The *bont* gene of the strains and the A5 subtype clustered into a branch. The evolutionary relationships with other A subtypes are shown in Figure 4.

### 4.4. Gene Cluster of the Neurotoxins, Antibiotic Resistance Genes, and Virulence Factors

The neurotoxin gene cluster type of *C. botulinum* strain was the “ha cluster”. The ha cluster contains two transcription units. Genes encoding neurotoxins and non-toxic-non-hemagglutinin (*ntnh*) are in a transcription unit, while genes encoding hemagglutinin (*ha70, ha17, and ha33*) are in another transcription unit. A5 (b3) strains contained the A5 gene, and a truncated B gene in a single ha cluster indicated a non-functional B toxin gene (Figure 3). Seven virulence genes *bont/a*, *fliP*, *glf*, *pseI*, *cloSI*, *htpB*, and *clpP* were identified by analyzing the JNPF1 sequencing results of *C. botulinum* using the VFDB database. Sequence identity analysis showed that bont/a was the major virulence gene. The CARD database was used to analyze the resistance genes, and the results showed that there were five resistance genes, namely *cfrC*, *lsaB*, *catB*, *vanZF*, and *mupB*. Sequence identity analysis showed that *cfrC* was the major resistance gene.

## 5. Discussion

Foodborne botulism poisoning incidents have occurred in many parts of the country, involving mostly fermented soy products and other high-protein foods in China. However, with the rapid development of prepared food, the risk of botulism caused by the consumption of vacuum-packaged foods has emerged. Vacuum-packaged foods may provide particularly favorable conditions for the growth of anaerobic, non-proteolytic *C. botulinum* and the formation of the botulinum neurotoxin [23]. Although numerous studies have confirmed that the BoNT produced by *C. botulinum* Group II (non-proteolytic) are more commonly found in vacuum-packaged frozen foods. It was found that the positive rate of *C. botulinum* in 74 vacuum-packaged vegetarian sausages from Finland and Germany was 32% with both Groups I and II strains, and BoNT genotypes B (33%), A (33%), and E (25%) were frequently detected [24]. Furthermore, an outbreak of *C. botulinum* subtype A poisoning occurred in Japan due to the ingestion of vacuum-packaged mustard lotus rhizomes in 1984 [25]. Therefore, improperly stored vacuum-packaged food can also contain the *C. botulinum* Group I strain.

In this study, the source of contaminated food was proved to be a vacuum-packaged spicy rabbit head. Epidemiological investigations showed that the spicy rabbit head came from Sichuan Province and was transported to Shandong Province in the cold chain by vacuum packaging from merchants. The epidemiological report showed that no cases of foodborne botulism were found after consuming spicy rabbit heads when they were fresh. Nevertheless, botulism symptoms occurred after the consumption of unheated spicy rabbit heads with the same batch that had been refrigerated for 6 months. The spicy rabbit heads were left at room temperature for three days before consumption, which might have provided suitable growth conditions for *C. botulinum* and BoNT production. The botulinum toxin can be completely destroyed by heating at 80 °C for 30 min or 100 °C for 10–20 min [26]. We recommend that vacuum-packed foods should be thoroughly heated before consumption to eliminate botulinum toxins. It is a risk factor that the spicy rabbit head had not undergone a formal vacuum-packaging process. Additionally, the prolongation of the preservation time leads to the depletion of oxygen in the vacuum self-packaged spicy head, which provided an anaerobic environment that promotes the growth and reproduction of *C. botulinum* spores and the secretion of the botulinum toxin. In addition, the preservation of spicy rabbit heads at room temperature for three days also accelerated the growth of *C. botulinum*. We hypothesized that this was the primary cause of this foodborne botulism outbreak.

The genomes of the *C. botulinum* strains were first discovered in 2007 [27]. Most of the genomes have a single gene encoding the botulinum neurotoxin [28]. A previous study by *Hutson* showed that the A(B) strain contains both *bont A* and *bont B*, but only the *bont A* gene is expressed and the botulinum serotype has been identified as type A by whole-genome sequencing [29]. A significant number of studies have reported that one strain possesses up to three neurotoxin genes and forms up to three types, namely A, B, and/or F neurotoxins [30]. Previous studies have confirmed that the BoNT gene is encoded by mobile genetic elements that can be transferred horizontally between the strains, which is thought to have contributed to the evolution of the BoNT locus, thus resulting in a large number of currently known emergences of different subtypes [31,32,33,34]. In our study, whole-genome sequencing was used for traceability analysis and strain typing. Core SNP analysis showed that the strains originated from the same strain. These results supported the epidemiological survey results, which provided a basis for traceability analysis in the beginning. We found that this botulism was caused by the consumption of a vacuum-packed spicy rabbit head contaminated with BoNT/A5(B3) produced by *Clostridium botulinum*. In this study, *Clostridium botulinum* sequence analysis confirmed that the genes encoding the A5 neurotoxin and the truncated B3 neurotoxin co-localized to the hemagglutinin complex (ha) containing the genes *ha70*, *ha17*, *ha33*, *botR, ntnh, and bont/A5*. *Clostridium botulinum* has a 76 bp deletion between the neurotoxin gene clusters *ha33* and *botR*, but this deletion does not affect A5 toxin production [35,36]. Meanwhile, the mouse toxicity test confirmed that the strains produced only type-A toxin but not a type-B toxin. However, the toxin gene cluster of *C. botulinum* A5 (b3) in this study was exactly the same as that of *C. botulinum* wound A5 (B3) from the UK [37]. However, the SNP analysis of the core genome showed that the *C. botulinum* strains were quite different from the reported *C. botulinum* A5 (B3) strains, and the differences in core SNPS were far from each other.

*C. botulinum* subtype A5 strains have been reported to cause many outbreaks of wound botulism and, rarely, foodborne botulism [38]. Two strains of the foodborne *C. botulinum* 1430-11 (A5B2) and 1141-11(A5B3) of the A5 genotype were identified in France. Strain 1430-11 (A5B2) was isolated from a case of botulism caused by the consumption of a commercial ready-to-eat product (pasta), whereas strain 1141-11 was isolated from the stool of a case of foodborne botulism of unknown origin [39]. This is the first report of foodborne botulism caused by the contamination of vacuum-packed spicy rabbit heads with *C. botulinum* type A5(b3). In addition, the virulence genes of *C. botulinum* strains in this event were mainly related to *bont/a*. This indicates that the main virulence of the strain is the type-A botulinum toxin. Studies have shown that the multidrug resistance gene *cfrC* is a methyltransferase gene that can mediate resistance to five classes of antibiotics. *cfrC* was the main drug resistance gene of *C. botulinum* strains in this outbreak event. A significant number of studies have reported that *cfrC* mediated by plasmids and chromosomes spreads horizontally through plasmids, insertion sequences, and transposons [40]. The multiple-drug resistance gene *cfrC* can spread among different bacterial strains, affecting the clinical treatment outcomes for humans and posing a threat to public health.

Foodborne botulism is a neurological disease with clinical manifestations distinct from other food poisonings and less gastrointestinal symptoms. The clinical symptoms of botulism are primarily neurological, which usually start with blurred vision and flaccid paralysis of the respiratory muscles or myocardium, which may occur in some severe cases [41,42]. Existing studies indicate that the rate of misdiagnosis in domestic hospitals in our country reaches 27.50% [17]. In this study, the case worsened due to successive misdiagnoses from three tertiary hospitals, thereby missing the optimal treatment opportunity. After a comprehensive assessment by the Qilu Medical Prevention Integration Mechanism, the diagnosis of foodborne botulism was confirmed through epidemiological investigation and laboratory tests. This indicated that the clinicians not only need to strengthen the training on foodborne botulism recognition but also accurately distinguish between botulism and similar diseases to ensure that patients receive the best treatment outcomes.

The clinical symptoms of the patient in this case were consistent with the characteristics of botulism and significant improvement was observed after treatment with the botulism antitoxin. Based on a comprehensive analysis of clinical symptoms, epidemiological findings, and laboratory testing results, we concluded that this outbreak was a case of foodborne botulism caused by BoNT/A5 (b3) from spicy rabbit heads. Our study underlines the risk of botulism from self-packaged vacuum meat products which might become a health concern for the public. Furthermore, we need to pay urgent attention to the food safety and hygiene of meat products purchased online. Appropriate measures must be taken, such as improving vacuum packaging or reducing storage temperatures, to minimize the possibility of exposure to pathogens or the production of the botulinum toxin throughout the food processing chain. Meanwhile, vacuum-packed food should be stored under appropriate conditions and kept within its shelf life.

## Figures and Tables

**Table 1:** Samples and test results.

Sample	MBA	HSFICA	Colloidal Gold Method	Real-Time PCR	Isolated Strains
Patient fecal	A	A	A	A and B	JNPF1 (*C. botulinum*)
Spicy rabbit head 1	A	A	A	A and B	JNSRH1 (*C. botulinum*)
Spicy rabbit head 2	A	A	A	A and B	JNSRH2 (*C. botulinum*)
TPGY culture of JNPF1	A	N/A	N/A	N/A	*C. botulinum*
TPGY culture of JNSRH1	A	N/A	N/A	N/A	*C. botulinum*
TPGY culture of JNSRH2	A	N/A	N/A	N/A	*C. botulinum*
Patient serum	N/A	A	A	N/A	N/A

Pangenomic analysis and SNP typing. Abbreviation: A = BoNT/A; A and B = BoNT/A and BoNT/B; N/A = No application.

## Data Availability

The data presented in this study are available upon request from the corresponding authors. The data are not publicly available due to privacy or ethical restrictions.
